# Effect of Waste Cooking Oil-Based Composite Materials on Radish Growth and Biochemical Responses

**DOI:** 10.3390/ma16237350

**Published:** 2023-11-25

**Authors:** Anita Staroń, Joanna Ciuruś, Magda Kijania-Kontak

**Affiliations:** 1 Department of Engineering and Chemical Technology, Cracow University of Technology, 24 Warszawska St., 31-155 Cracow, Poland; 2 Research Center for Cultivar Testing, A5 No. 9 St., 32-086 Węgrzce, Poland; 3 Department of Civil Engineering, Cracow University of Technology, 24 Warszawska St., 31-155 Cracow, Poland

**Keywords:** composite material, vegeblock, oil block, waste cooking oil, phytotoxicity, chlorophyll

## Abstract

Waste cooking oil poses a serious threat to human health and the environment, both in households and in larger communities. One of the applications of waste cooking oil is composite materials called vegeblocks, which can be used for construction purposes. These composites are formed by the process of polymerisation, esterification and polyesterification. The resulting materials exhibit mechanical strength in line with the requirements for paving blocks. Composite materials that have been annealed for a minimum of 20 h at 200 °C or higher have the highest tensile strength (above 5 MPa). In contrast, composites with the highest flexural strength were obtained after processing at 210 °C for 16 h. The Saxa 2 variety showed the greatest inhibition of storage root growth (almost 43% compared to the control sample), as well as stimulation of root and leaf blade growth (by a maximum of 61.5% and 53.5%, respectively, compared to the control sample). The composite obtained from the maximum process parameters resulted in significant growth of both the root and the green part of both radish varieties by up to 35%. The study showed that the presence of vegeblocks in the plants causes stress conditions, resulting in increased peroxidase content compared to the control sample. The presence of the oil composite in the soil did not increase the amount of catalase in the radish, and even a reduction was observed compared to the control sample.

## 1. Introduction

Waste cooking oil (WCO) is generated in food service outlets and households, and its improper storage poses a serious threat to human health and the environment [1,2]. During the frying process, various oxidation products are formed in the oil, such as polyglycerides and acyloglycerols, as well as dimeric and polymeric acids, which can increase the risk of cancer and cardiovascular diseases. Improper disposal of WCO poses an environmental threat by releasing these products into the ecosystem. Stringent regulations governing the recycling and disposal of cooking oil have led to an increase in WCO production, presenting challenges in effectively managing this issue [3]. To ensure environmental protection and food safety, the past decade has seen increased emphasis on recycling and reuse of WCOs [4,5]. Effective waste management is crucial not only to ensure healthy living conditions, but also to reduce the impact of climate change. As a result, strategies to increase the degree of circularity in waste management systems are being worked on intensively [6]. Waste vegetable oil is used in the production of, among other things, biofuels [7], lubricants, polyurethane foams, soaps, candles [8], pet food, bioplasticisers [9] and many others [10], and the industrial use of epoxidised vegetable oil is diversifying and rapidly commercialising [11]. Composite materials derived from waste cooking oil have also received a lot of attention recently. This is due to the simplicity of technology, cheap raw materials, and fitting in with the trend of environmentally friendly and cleaner technologies. 

The solid materials obtained from waste cooking oil have gained the name composite materials, vegeblocks, oil blocks, oil composites, solid oil materials or green materials, and can be used as building materials. A composite material is a material consisting of multiple phases, wherein reinforcing fillers are combined with a polymer matrix. This integration leads to synergistic mechanical properties that cannot be attained by either component alone [12]. Composite materials provide a versatile solution in the field of construction. Their unique mechanical properties, light weight and corrosion resistance make them widely used in various building structures. Composite materials make it possible to reinforce soils, create durable surfaces, and increase the insulation performance of buildings [13]. 

One of the major advantages of composite materials is their ease of adaptation to specific design requirements. By altering the type of reinforcement, the ratio of reinforcement to polymer matrix, and other parameters, it is possible to control the properties of composite materials and tailor them to specific applications [14]. Balo et al. have obtained building and insulation polymer materials from various oils, including linseed, rapeseed, palm and castor oils. These materials were characterised by low conductivity and low density, which consequently reduced their mechanical strength [15]. Waste cooking oil was used to degas an expanded polyester for potential use as a building material. The tensile and compressive strengths of this material were about 16 and 18 MPa, respectively [16]. Vu et al. obtained a material whose binder consisted of pure vegetable oil and glycerol and various types of ash as aggregate. The samples were annealed at 140–200 °C for 24–120 h. The most favourable temperature was 160 °C, and the compressive strength exceeded 30 MPa. The properties of the obtained materials were evaluated according to the British standards for concrete blocks and received a very favourable rating [17]. Composite materials were also obtained from both crude and used palm oil and aggregates (limestone from waste, bottom ash from incinerators and sand). Vegeblocks were cured at 160–170 °C for 12–48 h. An increase in mechanical strength was observed when the annealing time was extended beyond 24 h. In addition, it was shown that the material does not pose a fire hazard and does not ignite when exposed to 500 °C for 2 h [18]. Adebayo et al. [19] obtained a polymer binding material from vegetable oils. The composite materials were formed by annealing a mixture of sand and waste cooking oil at 160 to 200 °C, and their compressive strength was up to 34 MPa. The curing time could be reduced by adding sulfuric acid to the reaction mixture to act as a catalyst.

Optimisation of the oil-based polymer composite manufacturing process was presented by Staroń et al. [8]. Polymer materials containing waste cooking oil, catalyst and sand were produced at 180–220 °C for 12–20 h. The content of the catalysed oil in the reaction mixture was 15%, and the mass ratio of sulfuric acid (VI) to the catalysed oil was from 0.03 to 0.27. Even a 12 h annealing at 220 °C produced a composite material with high strength parameters. 

While numerous publications explore food oil-based composite materials’ potential as a construction resource, a conspicuous void exists regarding phytotoxicity assessments in real-world settings [20]. This omission is crucial for safeguarding both human well-being and the environment, as it is imperative to acknowledge the potential leaching of harmful substances from oil-based materials. The unpredictable atmospheric conditions and the presence of diverse compounds within the soil or precipitation can stimulate the release of composite material constituents. In field investigations, plants contend with an array of environmental variables, including fluctuating meteorological elements (temperature, humidity, sunlight and rainfall), along with fierce competition from other organisms. This approach offers a comprehensive understanding of how novel chemical substances impact plants within the intricate tapestry of natural settings, far surpassing the simplicity of laboratory conditions [21,22]. In nature, plants routinely interact with an assortment of other life forms, including microorganisms, insects, fungi, and competing producers. Field-based phytotoxicity studies provide a unique opportunity to factor in these intricate interactions, which wield a substantial influence on a substance’s toxicity to the ecosystem [23,24].

This article delineates the repercussions of integrating waste cooking oil-based composite materials, colloquially known as “vegeblocks,” into the soil on the growth, enzyme levels, and assimilation pigments of two radish varieties: *Raphanus sativus* L. *var. sativus* (De dix-huit jours) and *Raphanus sativus sativus* L. *var. sativus* (Saxa 2). Notably, these experiments were conducted under uncontrolled field conditions, allowing the composite materials to undergo rigorous exposure to external environmental factors.

## 2. Research Methodology

### 2.1. Production of Vegeblocks

Composite materials were obtained by heating rapeseed oil, which underwent thermal food processing (Waste Cooking Oil, WCO) with sulfuric acid, and quartz sand with a fraction of 0.5–1.4 mm, in a dry-air state. WCO was mixed with sulfuric acid, followed by the addition of sand. The mixture was homogenised for 5 min in a planetary mixer, and then transferred to aluminium moulds and compacted on a vibrating table. The samples were heated at temperatures ranging from 190 to 210 °C for a duration of 16 to 20 h. The content of catalysed oil (CO) and natural additive was counted in relation to the weight of sand. The vegeblocks exhibited different levels of catalysed oil content and varying mass ratios of the acid catalyst to the catalysed oil (Table 1).

The thermal analysis of the oil was conducted using the differential scanning calorimetry and thermogravimetric analysis SDT 650 DSC/TGA apparatus from TA Instruments (New Castle, DE, USA) in the temperature range of 25–1000 °C with a heating rate of 10 °C/min. The molecular structure analysis of WCO and composites was performed using Fourier-transform infrared spectroscopy with the Nicolet iS5 FT-IR spectrometer from Thermo Scientific (Waltham, MA, USA) in the wavelength range of 500–4000 cm^−1^.

The surface structure of the vegeblocks was observed using the scanning electron microscope Hitachi TM-3000 (Hitachi-hightech, Tokyo, Japan) equipped with an energy-dispersive X-ray microanalyser (EDS). The mechanical strength tests were carried out using the Zwick-Roell Z600 testing machine (Zwick Roell, Ulm, Germany) (initial force of 25 N, testing speed of 1 kN/min).

To assess the influence of the mass ratio of sulfuric acid to waste cooking oil, two mixtures were analysed. The ratios were as follows: 0.02 (sample A), 0.08 (sample B). The FT-IR analysis was performed after 19 h of storing the mixtures at room temperature. Additionally, samples that had the same composition were cured at 200 °C for 19 h (samples A-200, B-200). FT-IR analysis of those materials was performed after cooling them down.

Additionally, in order to define number average molecular weight (Mn), average molecular weight (Mw) and dispersity (D), gel permeation chromatography (GPC) analysis was performed using a Knauer chromatograph (Knauer, Berlin, Germany) equipped with a PLgel MIXED-E column (Agilent, Santa Clara, CA, SUA). In order to perform the calibration, polystyrene standards were used. Tetrahydrofuran was applied as an eluent (rate was equal to 1 mL/min at room temperature).

To determine the leachability of heavy metals and polycyclic aromatic hydrocarbons (PAHs) from vegeblocks, the material was crushed into approximately 1 cm pieces, placed in deionised water at a mass ratio of 1:20, and shaken for 7 days. Heavy metals (copper, iron, magnesium, tin and zinc) were determined in the filtrate using atomic absorption spectroscopy with the Perkin-Elmer 370 spectrophotometer (PerkinElmer, Waltham, MA, USA). The determination of PAHs was performed using the LC-ESI-MS/MS method.

### 2.2. Phytotoxicity Studies

Phytotoxicity studies were conducted using two varieties of radish: *Raphanus sativus* L. *var. sativus* (De dix-huit jours) and *Raphanus sativus* L. *var. sativus* (Saxa 2). These plants were chosen due to their relatively short vegetation period (approximately 25 days), well-known plant morphology and the ability of all their parts to be consumed by humans and animals. The experiments were carried out under field conditions in a south-facing location on brown soil. The selected soil had never been cultivated before, which helped to avoid the effects of soil fatigue and residual fertilisers or pesticides. Prior to sowing, the soil was loosened. The seeds were sown in rows 3.5 cm apart to allow for the unrestricted development of roots. Each row contained a vegeblock with a different composition, and the spacing between the rows prevented the intermingling of leached components from the oil block. Control samples were also prepared, consisting of rows without oil blocks. The rows were exposed to atmospheric conditions, and the soil moisture was regulated by manual watering with tap water.

The effect of oil blocks on the development of *Raphanus sativus* was determined by evaluating morphological traits, i.e., leaf rosette foliage, leaf length, and length of thickening. Assimilation pigments and enzyme contents of the plants were examined. Catalase activity was measured by the UV spectrophotometric method, which involves monitoring the change in absorbance at 240 nm for high concentrations of hydrogen peroxide solution [25]. Determination of peroxidase in *Raphanus sativus* leaves was carried out according to the methodology in [26]; absorbance of the solution was measured at 420 nm.

The content of assimilatory pigments (chlorophyll and carotenoids) was determined at 470, 647 and 664 nm after sample preparation according to the procedure proposed by Oren et al. [27].

Pareto charts of the effects were prepared, where the effect ratings obtained using the ANOVA procedure were arranged in descending order of their absolute values. The magnitude of each effect is represented by a bar and a line indicating the threshold for statistical significance [28]. The statistical analysis was performed using version 10 of STATISTICA by StatSoft^®^ [29].

## 3. Results and Discussion

### 3.1. Vegeblocks

**Figure 1:**
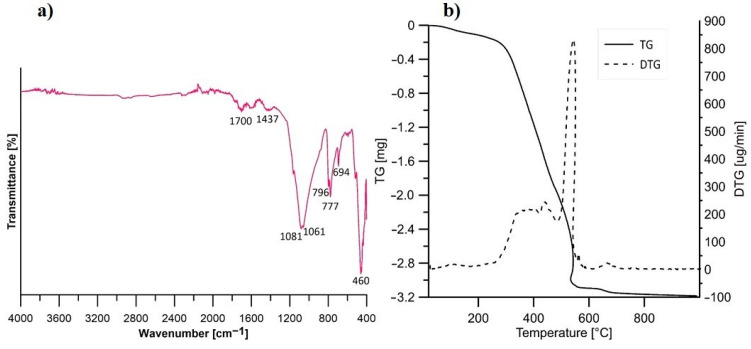
(**a**) FT-IR spectrum and (**b**) TG/DTA spectra of vegeblock No. 10.

The FT-IR spectrum of vegeblock No. 10 (Figure 1a) shows bands around the wavenumber 1700 cm^−1^, which can be attributed to the stretching vibrations of the C=O ester group. Bands at 1437 cm^−1^ correspond to bending vibrations of the C–H in alkanes, while the range of 1080–1060 cm^−1^ is associated with stretching vibrations of the C–O bonds [30]. Deformation vibrations of C–H bonds are present at wavenumbers 777–796 cm^−1^. Peaks observed at a wavenumber of 694 cm^−1^ are related to bending vibration of O–Si–O [31], while those around 460 cm^−1^ are associated with torsional motion in organic molecules [32]. Figure 1b presents the results of thermogravimetric/differential thermal analysis (TG/DTA) of vegeblock No. 10. The initial mass loss of the oil block occurred in the temperature range from 25 °C to 100 °C. This is mainly due to the loss of absorbed water from the surrounding environment [33]. The highest mass loss associated with sample decomposition is observed in the temperature range of 250–600 °C, and above this temperature, the sample mass stabilises. In the temperature range of 200–350 °C, the polyesters undergo decomposition [34]. The degradation at around 600 °C can be attributed to the burning of the carbonaceous residues [35].

The presence of heavy metals such as cadmium, lead, iron, arsenic and mercury was not identified in the leachate after vegeblock incubation. Table 2 shows the polycyclic aromatic hydrocarbon (PAH) content of the leachates after incubation of the composites. For none of the seeps did the sum of PAHs exceed 0.01 mg/L, which is in accordance with Council Directive 98/83/EC of 3 November 1998 on the quality of water intended for human consumption.

**Figure 2:**
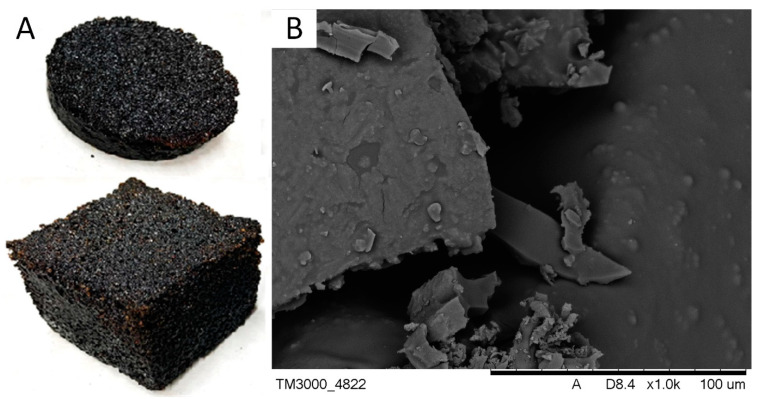
(**A**) A photograph and (**B**) SEM micrograph of vegeblock No. 8.

Figure 2 shows a photograph and an SEM micrograph of vegeblocks produced at 200 °C in which the ratio of acid catalyst to catalysed oil was 0.14 (sample No. 8). The materials were characterised by high porosity and sharp edges but varied in mechanical strength.

**Figure 3:**
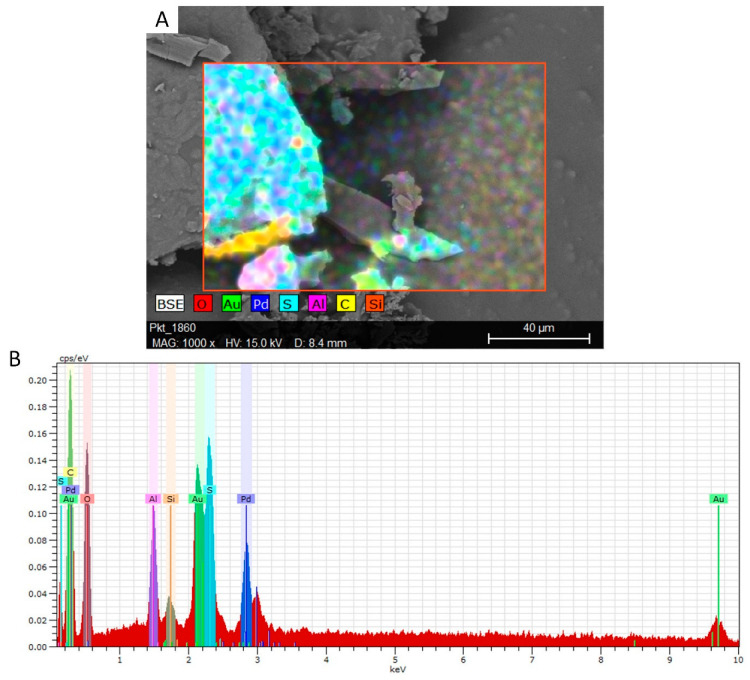
(**A**) SEM micrograph and (**B**) EDX map of vegeblock No. 8.

Silicon and aluminium from the aggregate, sulfur from the acid catalyst, carbon from the WCO component and gold and palladium from the sputtering of the sample were identified on the surface. Figure 3 shows the SEM micrograph and EDX map of vegeblock No. 8.

**Figure 4:**
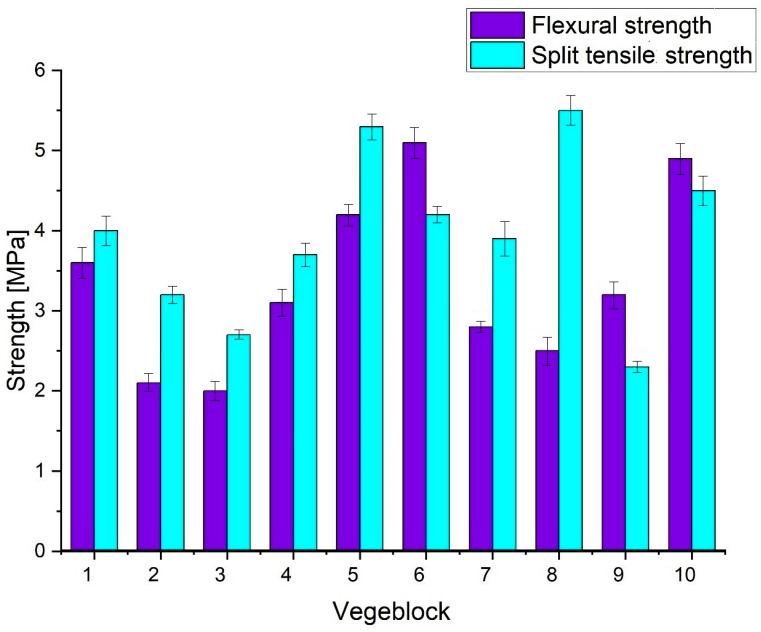
Flexural and split tensile strength of vegeblocks No. 1 through No. 10.

The obtained composites exhibited the mechanical strength required for paving blocks [36]. The vegeblock No. 2, obtained at 190 °C for 18 h, exhibited the lowest tensile strength at splitting, with a value of approximately 2.3 MPa. On the other hand, the highest tensile strengths were observed in vegeblocks No. 3 and No. 6 (above 5 MPa), which were obtained by heating for 20 h at temperatures of 200 and 210 °C, respectively (Figure 4).

The mechanical strength properties of the obtained vegeblocks can be compared with those of a layered composite made from polyester resin and kenaf fiber [37]. In terms of flexural strength, a similar trend was observed. Vegeblocks No. 1 and No. 7, characterised by a flexural strength of approximately 5 MPa, were obtained at a temperature of 210 °C for 16 h. On the other hand, vegeblock No. 8, which exhibited the lowest flexural strength, was obtained at a temperature of 190 °C for 17 h. The vegeblocks with the lowest mechanical strength also had the highest mass ratio of the acidic catalyst to the catalysed oil. This higher ratio led to faster polymerisation of the samples and their curing at a lower temperature. As a result of heating catalysed vegetable oil mixed with aggregates by 190 °C for 24 h, Eco-Friendly Vege Roofing Tiles with flexural strength up to 9.5 MPa were also obtained [38]. In comparison, a composite of waste cooking oil whose matrix contained asbestos fibers obtained at 220 °C for 12 h had a compressive ns strength of 28.3 MPa [39]. 

**Figure 5:**
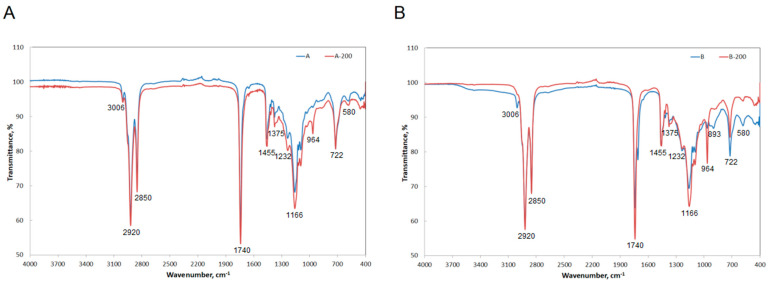
FTIR spectra of waste cooking oil and sulfuric acid mixtures mixed and stored at room temperature and cured at 200 °C: (**A**) sample A and A-200, (**B**) sample B and B-200.

The exact spectra comparing the curves obtained before and after curing each sample are presented in Figure 5. In the case of samples mixed and stored at room temperature, one may observe the characteristic peaks. In general, bands at 2920 and 2850 cm^−1^ originate from –C–H (CH_2_) stretching asymmetric vibrations and –C–H (CH_2_) stretching symmetric vibrations, respectively, which are located in the aliphatic structures in the chain backbone. Bands at 1740 cm^−1^ may be attributed to stretching vibrations of –C=O bonds in the ester groups. Bands that are found in the region between 1375 and 1232 cm^−1^ correspond to C–O stretching vibrations. O–H bending vibrations are confirmed by bands in the range of 1455–1407 cm^−1^.

When analysing spectra comparing the curves obtained before and after hardening of each sample, attention should be paid to peaks that disappear and appear after hardening of the reaction mixtures at a temperature of 200 °C. It may be seen that the following peaks disappear: 3006, 1375, 1166, 1044, 910 and 893 cm^−1^. These peaks are attributed to the following groups: =C–H (cis) stretching vibration, –C–H (CH_3_) bending (sym), –C–O stretching and –CH_2_ bending vibrations, –C–O stretching vibrations, –HC=CH (cis) bending out of plane and C=CH_2_ wagging vibration, respectively. On the other hand, one may distinguish peaks that appear. These are 1592, 1363 and 964 cm^−1^. They may be attributed to the following characteristic groups: –COO– stretching of carbonyl groups, OH bending vibrations and –HC=CH (trans) bending out of plane, respectively [40]. 

**Figure 6:**
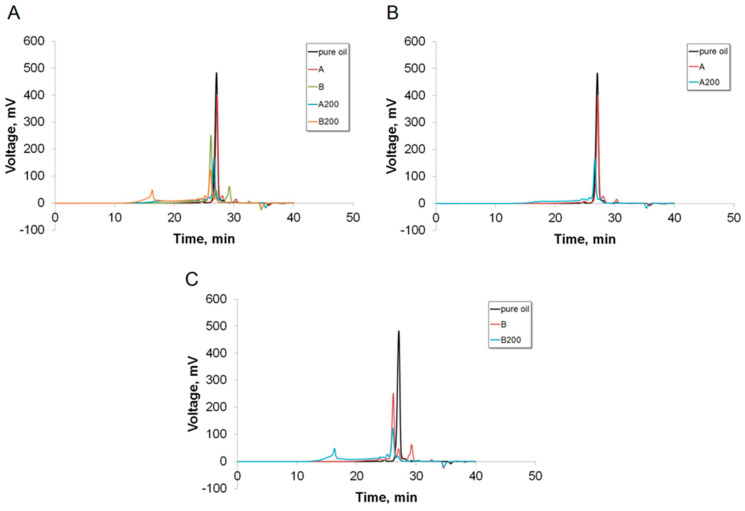
Results of GPC analysis of: (**A**) WCO sample, (**B**) WCO and sulfuric acid mixture, (**C**) mixtures cured at 200 °C.

The results of GPC analysis presenting the formation of individual components are shown in Figure 6. The characteristic values defined based on GPC are shown in Table 3. N, testing speed of 1 kN/min).

To assess the influence of the mass ratio of sulfuric acid to waste cooking oil, two mixtures were analysed. The ratios were as follows: 0.02 (sample A), 0.08 (sample B). The FT-IR analysis was performed after 19 h of storing the mixtures at room temperature. Additionally, samples that had the same composition were cured at 200 °C for 19 h (samples A-200, B-200). Samples of waste cooking oil with the mass ratio of sulfuric acid to waste cooking oil of 0.02 (sample A) and 0.08 (sample B) were analyzed 19 hours after homogenization at room temperature and after curing at 200 °C for 19 h (samples A-200, B-200).

The molecular weight of oil is equal to 620 g/mol. Peaks appearing to the left of the pure oil peak indicate the formation of smaller molecules, which may be caused by hydrolysis of the oil. However, after curing the reaction mixtures at 200 °C, greater molecules are formed, which is indicated by appearance of peaks on the right of the pure oil peak. Their molecular weight is two times bigger than before curing. Their formation may be attributed to the oligomerisation of individual units. Also, esterification of shorter molecules is possible—this is confirmed by formation of a –COO– group, which was indicated during FTIR analysis.

### 3.2. Phytotoxicity Studies

It was observed that in the case of radish cultivar Saxa 2, vegeblocks had a more stimulating effect on plant development than in the case of cultivar De dix-huit jours when compared to the control samples. The highest inhibition of storage root length growth of almost 43% compared to the control sample was observed for the Saxa 2 variety grown in soil with vegeblock No. 8. Under the same conditions, the storage root of the De dix-huit jours radish was stimulated to grow (more than 10% increase). Certain bio-based plastics were found to suppress root growth while promoting shoot growth in dicotyledonous plants [41]. A clear stimulation of the Saxa 2 variety was evident under the influence of composite Nos. 2, 3, 4, 5, and 9 through 10. This was particularly true for the thickness of the radish root (a maximum of 61.5% over the control sample) and the length of the leaf blade (53.5% over the control sample).

**Figure 7:**
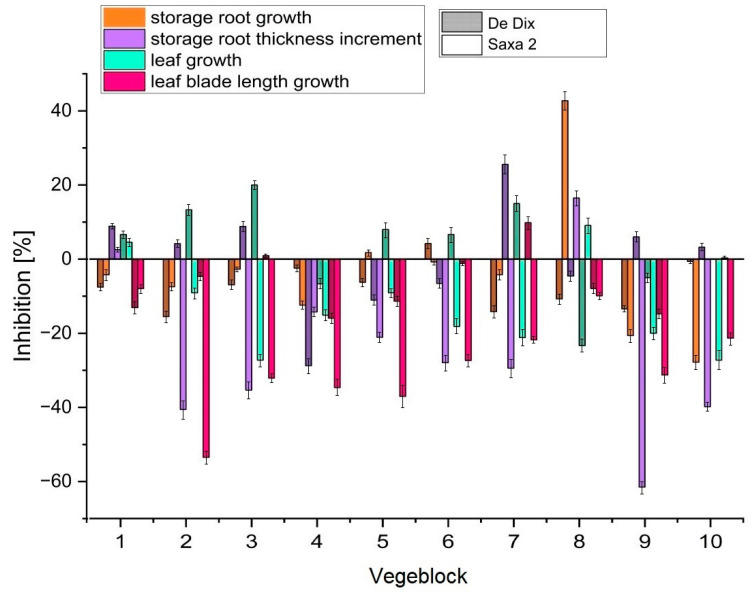
Inhibition of leaf and storage root growth in De dix-huit jours and Saxa 2 radish varieties when compared to the control radish samples.

The lowest effect on the simultaneous development of the aboveground and underground parts of radishes (up to ±13%) was exerted by vegeblock No. 1. This may be due to the fact that it had the lowest content of catalysed oil. In addition, composites obtained at high temperature and extended time had a more frequent stimulating effect on plant growth. Composite No. 4, obtained at maximum process parameters, resulted in growth of the root as well as the green part of both radish varieties by up to 35% (Figure 7). Similar observations apply to the effects of biodegradable and non-biodegradable plastics on monocotyledonous and dicotyledonous plants. The non-biodegradable substance was polypropylene of petroleum origin. The presence of plastics had no effect on the germination of seeds of higher plants but did affect their growth. The observed effects varied from inhibition to stimulation of growth. Dicotyledonous plants proved to be particularly sensitive to the presence of plastics, which was evident in the length of the root, as well as the stem, and importantly, this phenomenon occurred for every material tested. Inhibition of plant root growth of up to 22% was observed when plants were exposed to polyhydroxybutyrate and polylactide-based plastics [42]. Studies with soybeans have shown a significant negative effect of polyethylene on the height of this plant and the diameter of the stem during the entire growth stage of soybeans. Leaf area was reduced up to more than 11% compared to the control sample [43]. Numerous polymers have been demonstrated to enhance soil shear strength, enhance volumetric stability, enhance water retention, and prevent erosion even at very low soil concentrations, which directly influences plant growth [44].

**Figure 8:**
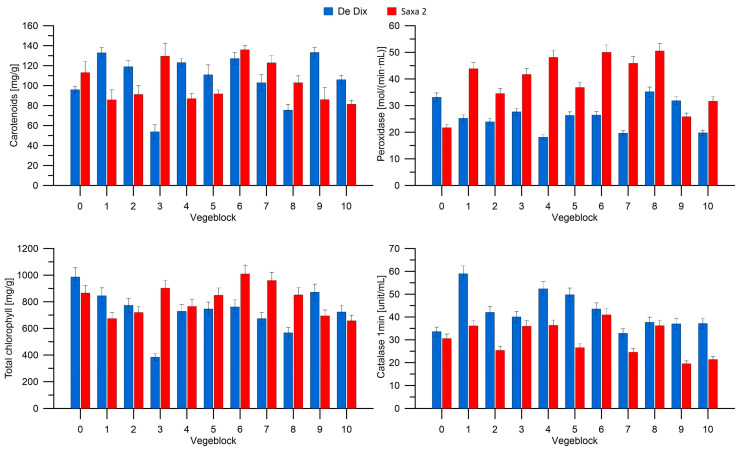
Carotenoids, peroxidase, total chlorophyll, and catalase content in De dix-huit jours and Saxa 2 radish varieties.

Figure 8 presents carotenoids, peroxidase, total chlorophyll, and catalase content in De dix-huit jours and Saxa 2 radish varieties. There was some regularity observed in the enzymes present in radishes. In most cases, the content of peroxidase was higher in the Saxa 2 variety, while catalase was higher in the De dix-huit jours variety. The highest content of peroxidase was found in the Saxa 2 radish grown in the presence of composite Nos. 1, 4, 6 and 8. The presence of each block caused stressful conditions, resulting in an increased content of this enzyme compared to the control. The increased content of peroxidase can be a response of the plant to oxidative stress, which arises from the excessive accumulation of reactive oxygen species (ROS) due to various factors, such as UV radiation, environmental pollution, high temperature, water deficiency, etc. Peroxidase is involved in the reduction and neutralisation of ROS, thus protecting the plant from oxidative damage. It can be speculated that, in this case, the increased content of peroxidase is a sign of the plant’s adaptation to new environmental conditions or an attempt to neutralise toxic substances present in the environment. The surprising aspect in the case of the De dix-huit jours variety is that the presence of vegeblocks in the soil did not lead to an increase in the amount of peroxidase in the plant, and even a significant decrease was observed (in the case of composite Nos. 4, 7 and 10).

The content of catalase in the plants also varied. In the case of the Saxa 2 variety, the presence of the vegeblock in the soil did not result in an increase in the amount of this enzyme in the radish. For plants grown in the vicinity of composite Nos. 2, 5, 7, 9 and 10, a reduction in the amount of catalase was observed compared to the control. Tsouvaltzis and Brecht described the influence of stress factors on changes in the quality and activity of antioxidant enzymes in radish during storage at temperatures of 5 or 10 °C. The research results indicate that plants stored at lower temperatures had a longer shelf life, lower water loss, higher levels of nutrients (such as vitamin C), and higher activity of antioxidant enzymes, including peroxidase [45].

The observed changes in chlorophyll content indicate that the presence of certain oil-based blocks in the soil induces oxidative stress. A strong response of the De dix-huit jours radish variety to the presence of vegeblock No. 3 in the soil is evident, as the total chlorophyll content decreased by approximately 60% compared to the control. The Saxa 2 variety shows a lesser degree of chlorophyll content change in response to the presence of oil materials, and a slight increase in chlorophyll content may be associated with favourable growth conditions, adequate light exposure or nutrient availability. Another parameter describing proper plant development is the ratio of chlorophyll a to chlorophyll b (chl a/chl b) and the total chlorophyll to carotenoid ratio. Slight changes in the chl a/chl b ratio are visible in the case of the Saxa 2 variety, indicating the presence of oxidative stress in radishes grown in soil with oil-based composites. The decrease in carotenoid content may suggest damage to the photosystems and disturbances in their functioning. In the case of the De dix-huit jours variety, a two-fold increase in the chl a/chl b ratio compared to the control is observed, which is caused by oxidative stress. Additionally, an increase in the carotenoid content relative to the total chlorophyll content indicates a certain activation of the plants prior to this stress. These relationships were presented by Gengmao using safflower as an example [46]. He evaluated changes in various aspects, such as plant growth and antioxidant enzymes, providing information on the mechanism through which safflower adapts to stress.

### 3.3. Evaluation of the Significance of Effects

**Figure 9:**
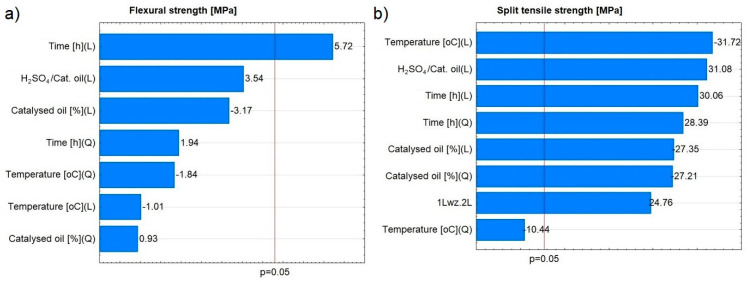
Pareto charts of the effects for (**a**) flexural strength and (**b**) split tensile strength.

In the case of flexural strength, the only significant effect was the linear effect associated with the variable of time. For tensile strength, the quadratic effect associated with this variable was the only non-significant effect (Figure 9). Ardila-Suárez et al. [47] also re-ported the effect of sulfuric acid as a catalyst on the polymer compound formation reaction. They observed the process of polyglycol polymerisation in a combinatorial experiment, examining the effect of temperature and catalyst concentration. As in the case of composites based on waste cooking oil, it was possible to modify their physicochemical properties by controlling process parameters. 

**Figure 10:**
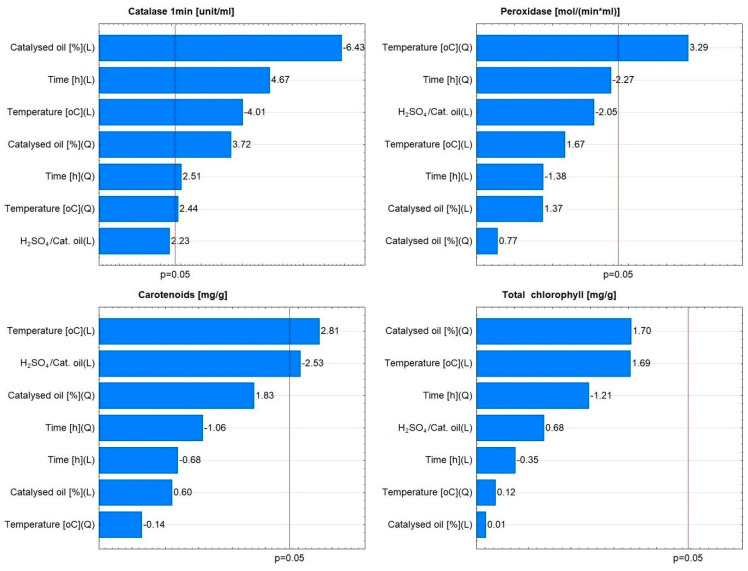
Pareto charts of the effects for catalase, peroxidase, carotenoids, and total chlorophyll for De dix-huit jours variety.

**Figure 11:**
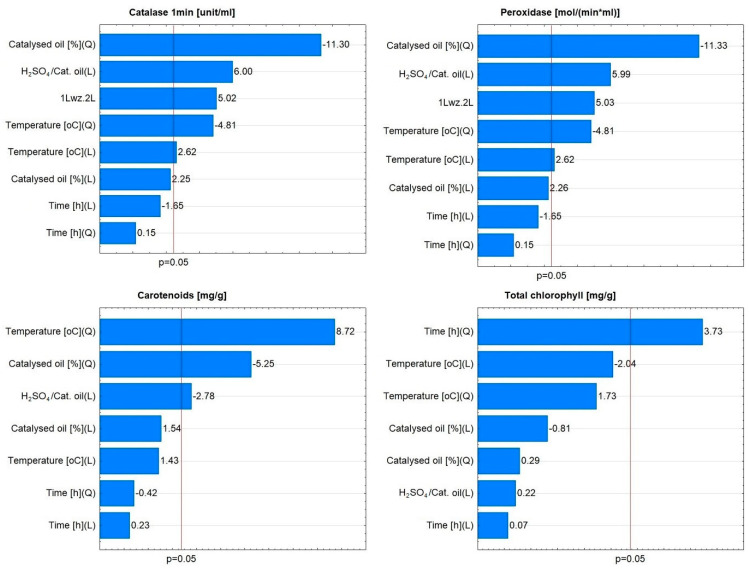
Pareto charts of the effects for catalase, peroxidase, carotenoids, and total chlorophyll for Saxa 2 radish variety.

No relationship was observed between the radish variety and significant effects on assimilation pigments and enzymes. For example, in the case of the De dix-huit jours variety, the linear effect related to the variable H_2_SO_4_/catalysed oil was found to be non-significant (Figure 10). For Saxa 2, the linear effect related to the variable H_2_SO_4_/catalysed oil, the quadratic effect related to the variable catalysed oil [%] and the interaction effect of both variables, as well as the linear and quadratic effects related to the variable temperature [°C], were found to be significant (Figure 11). 

It should be emphasised that due to the field conditions of the study, the system was subjected to the influence of natural factors whose strength is impossible to determine. Both varieties were grown at the same time, allowing for a comparison of the level of plant development between them. The influence of controlled factors (temperature of obtaining composite materials, duration of their heating, mass ratio of acidic catalyst to catalysed oil) is not solely the effect of individual factors but actually the result of their interactions with external factors.

Protecting the ecosystem and raising living standards require proper waste management. Today, every human activity generates waste, which entails the implementation of environmentally friendly and economically efficient measures. Therefore, it is crucial to optimise the use of resources and production processes in the construction sector, promote sustainable development and use advanced sustainability assessment tools, including life cycle assessment and exergy [48,49].

## 4. Conclusions and Future Perspectives

The obtained composite materials demonstrated satisfactory mechanical strength, meeting the requirements for paving blocks. The highest tensile strength (above 5 MPa) was observed in vegeblocks heated for at least 20 h at a temperature of 200 °C or higher, while the highest flexural strength was achieved at 210 °C for 16 h.

The composite materials had a more stimulating effect on the growth of the Saxa 2 variety of radishes compared to De dix-huit jours. The greatest inhibition of the growth of storage root length was observed in the Saxa 2 variety, reaching nearly 43% compared to the control group. Additionally, there was a stimulation of root thickness (up to 61.5% compared to the control) and leaf blade length (53.5% compared to the control). The presence of each composite material induced stressful conditions in the plants, resulting in an increased content of peroxidase compared to the control group. The content of catalase in the plants also varied. In the case of the Saxa 2 variety, the presence of vegeblocks in the soil did not increase the amount of this enzyme in radishes, and even a reduction in catalase content was observed compared to the control group. 

The process of producing composites from waste cooking oil faces several significant constraints, including limited scale of production due to available furnace space and availability of raw materials (geopolitical situation). Another challenge is the variation in the composition of used cooking oils. Waste cooking oils can have different compositions, which in turn can affect the quality and properties of the final composites. All of these limitations make the production of composites from waste cooking oil a challenge that requires attention and management to benefit both the environment and the economy.

The use of waste oil in the production of building materials is in line with the principles of “green chemistry.” However, despite the developed description of plant block manufacturing processes, there is still a lack of comprehensive research on the environmental impact of these products and environmental safety issues. 

The presented research results indicate complex interactions between the components of the composite materials, soil and plant varieties. Given that these composites can serve as building materials and will come into contact with soil, there is a strong need for further research into optimising the manufacturing process of these materials to minimise their environmental impact.

## Figures and Tables

**Table 1:** Parameters of composite material production process.

Sample	CO [%]	H_2_SO_4_/CO [g/g]	T [°C]	t [h]
1	20.0	0.240	210	18.0
2	25.0	0.240	190	18.0
3	22.5	0.140	200	20.0
4	25.0	0.240	210	20.0
5	22.5	0.240	190	19.0
6	25.0	0.240	210	20.0
7	22.5	0.240	210	16.0
8	22.5	0.240	190	17.0
9	22.5	0.240	210	19.0
10	22.5	0.240	200	19.0

**Table 2:** The content of polycyclic aromatic hydrocarbons in leachates after vegeblock incubation.

PAH ng/mL	Vegeblock No. 2	Vegeblock No. 9
naphthalene	0.130 ± 0.001	0.211 ± 0.011
acenaphthylene	0.00530 ± 0.0001	0.00820 ± 0.00031
acenaphthene	0.0114 ± 0.0009	0.0385 ± 0.0015
fluorene	0.0221 ± 0.0010	0.0492 ± 0.0019
phenanthrene	0.111 ± 0.017	0.198 ± 0.006
anthracene	0.0251 ± 0.0024	0.0391 ± 0.0022
fluoranthene	0.0396 ± 0.0032	0.0584 ± 0.0022
pyrene	0.0265 ± 0.0014	0.0388 ± 0.0018
benz(a)anthracene	0.00710 ± 0.00037	0.00550 ± 0.00030
chrysene	0.0132 ± 0.0008	0.00320 ± 0.00024
benzo(b)fluoranthene	0.0132 ± 0.0011	0.00410 ± 0.00026
benzo(k)fluoranthene	0.00520 ± 0.00018	<0.00176 ± 0.00011
benzo(a)pyrene	0.00390 ± 0.00020	<0.00219 ± 0.00015
indeno(1,2,3-cd)pyrene	0.00360 ± 0.00019	<0.000850 ± 0.000022
dibenzo(ah)anthracene	0.00210 ± 0.00011	<0.000950 ± 0.000031
benzo(ghi)perylene	0.00420 ± 0.00017	<0.00119 ± 0.00012
Sum	0.423	0.654

**Table 3:** Parameters of components defined based on GPC analysis.

	Mn [g/mol]	Mw [g/mol]	D
Oil	620	632	1.02
A	577	659	1.14
A200	987	3328	3.37
B	608	887	1.46
B200	1542	9029	5.85

## Data Availability

Data are contained within the article.

## Abbreviations

WCO	waste cooking oil
CO	catalysed oil
T	temperature
t	time
DSC	differential scanning calorimetry
TGA	thermogravimetric analysis
TA	thermal analysis
FT-IR	Fourier-transform infrared spectroscopy
SEM	scanning electron microscope
EDS	energy-dispersive X-ray spectroscopy
Mn	number average molecular weight
D	dispersity
GPC	gel permeation chromatography
Mw	average molecular weight
PAH	polycyclic aromatic hydrocarbon
LC-ESI-MS/MS	liquid chromatography-electrospray ionisation-tandem mass spectrometry
ANOVA	analysis of variance
chl a	chlorophyll a
chl b	chlorophyll b
